# Interventions for sustained healthcare professional behaviour change: a protocol for an overview of reviews

**DOI:** 10.1186/s13643-016-0355-9

**Published:** 2016-10-13

**Authors:** Stephan U. Dombrowski, Pauline Campbell, Helen Frost, Alex Pollock, Julie McLellan, Steve MacGillivray, Anna Gavine, Margaret Maxwell, Ronan O’Carroll, Helen Cheyne, Justin Presseau, Brian Williams

**Affiliations:** 1 Division of Psychology, Stirling University, Cottrell Building, Room 3A107, Stirling, FK9 4LA UK; 2 Nursing, Midwifery and Allied Health Professions Research Unit (NMAHP RU), Glasgow Caledonian University, Glasgow, UK; 3 Scottish Improvement Science Collaborating Centre, Nursing, Midwifery and Allied Health Professions Research Unit (NMAHP RU), University of Stirling, Stirling, UK; 4 School of Health Sciences, University of Stirling, Stirling, UK; 5 Evidence Synthesis Training and Research Group, University of Dundee, Scotland, UK; 6 Centre for Practice-Changing Research, Ottawa Hospital Research Institute, Ottawa, Canada

**Keywords:** Overview, Sustainability, Professional behaviour change, Interventions, Healthcare

## Abstract

**Background:**

Failure to successfully implement and sustain change over the long term continues to be a major problem in health and social care. Translating evidence into routine clinical practice is notoriously complex, and it is recognised that to implement new evidence-based interventions and sustain them over time, professional behaviour needs to change accordingly. A number of theories and frameworks have been developed to support behaviour change among health and social care professionals, and models of sustainability are emerging, but few have translated into valid and reliable interventions. The long-term success of healthcare professional behavioural change interventions is variable, and the characteristics of successful interventions unclear. Previous reviews have synthesised the evidence for behaviour change, but none have focused on sustainability. In addition, multiple overlapping reviews have reported inconsistent results, which do not aid translation of evidence into practice. Overviews of reviews can provide accessible succinct summaries of evidence and address barriers to evidence-based practice. We aim to compile an overview of reviews, identifying, appraising and synthesising evidence relating to sustained social and healthcare professional behaviour change.

**Methods:**

We will conduct a systematic review of Cochrane reviews (an Overview). We plan to systematically search the Cochrane Database of Systematic Reviews. We will include all systematic reviews of randomised controlled trials comparing a healthcare professional targeted behaviour change intervention to a standard care or no intervention control group. Two reviewers will independently assess the eligibility of the reviews and the methodological quality of included reviews using the ROBIS tool. The quality of evidence within each comparison in each review will be judged based on the GRADE criteria. Disagreements will be resolved through discussion. Effects of interventions will be systematically tabulated and the quality of evidence used to determine implications for clinical practice and make recommendations for future research.

**Discussion:**

This overview will bring together the best available evidence relating to the sustainability of health professional behaviour change, thus supporting policy makers with decision-making in this field.

## Background

New clinical evidence, technologies and medicines continue to be developed, promising to improve the health and well-being of populations. Production of such evidence does not guarantee its implementation, let alone its sustained use. Healthcare professionals often need to adapt their clinical or caring behaviours to introduce new evidence-based practice. Supporting healthcare professionals to modify their behaviour and subsequently maintain this change requires effective, replicable implementation interventions that promote sustainable change to ensure consistent delivery of the best clinical practice.

**Fig. 1:**
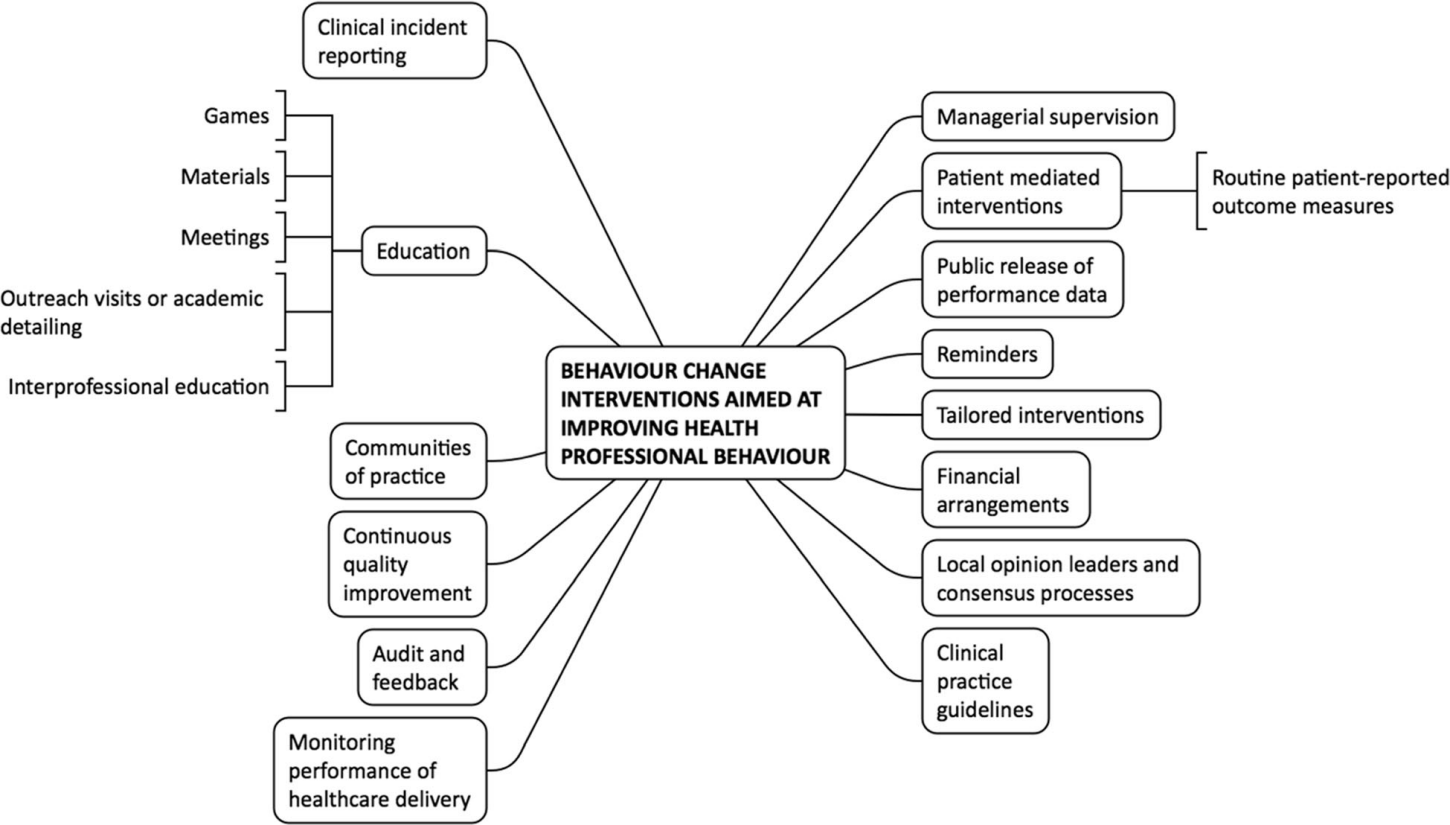
Examples of behaviour change interventions targeting health professional behaviour (adapted from [1])

Evidence synthesised within multiple systematic reviews has demonstrated that interventions can be effective in changing healthcare professional practice. A wide range of behaviour change interventions can be delivered to change healthcare professional behaviour (Fig. 1). These interventions can be described according to broad strategies, such as those outlined by Cochrane Effective Practice and Organisation of Care (EPOC) group [1] and the ERIC project [2], although various different descriptive frameworks exist [3].

Several reviews have uncovered evidence of intervention strategies that have positively influenced healthcare professional behaviour [4–9]. For example, Ivers et al. [4] found that audit and feedback significantly influenced the performance of healthcare professionals responsible for patient care. They found small, but potentially important, improvements in professional practice. Similarly, O’Brien et al. [10] examined the effectiveness of educational outreach visits tested in 69 studies (using qualitative and quantitative designs) involving more than 15,000 healthcare professionals. They found consistent and small effects on prescribing practices, with effects on other types of professional performance varying from small to modest improvements. Other systematic reviews investigated behaviour change interventions within a specific area of healthcare practice such as weight management [5], device-related infections [11] and antibiotic prescribing [7, 8].

More recently, Johnson and May 2015 [12] carried out a systematic overview of behaviour change interventions, using a theory-led analysis. Within 67 reviews, they found that a number of interventions, such as modifying peer group norms and expectations and relational restructuring, may be effective in changing healthcare professional behaviour but they did not focus on interventions sustained over the long term.

Despite many systematic reviews indicating promising results of healthcare professional behaviour change interventions in several areas, long-term effects have generally not been studied or evaluated systematically. This is an important oversight since even relatively small changes in provider behaviour, if maintained, have the potential to beneficially impact behaviour and health at a population level. Therefore, there is a need to systematically explore the long-term effectiveness of healthcare professional focused behaviour change interventions (i.e. their sustainability) and to identify which components of these interventions are key factors in effecting any sustained changes.

### Sustainability

“Sustainability” is a multidimensional concept that can refer to programmes, capacity, outcomes and practice, but it is poorly defined in the literature [13, 14]. A systematic review that examined sustainability of new programmes and innovation reported that few authors present a working definition or guidance for a model of sustainability [14].

Scheirer [15] describes three definitional measures of sustainability: (a) continuing to deliver beneficial services/outcomes to clients (an individual level of analysis); (b) maintaining the programme and/or its activities in an identifiable form, even if modified (an organizational level of analysis) and (c) maintaining the capacity of a community to deliver programme activities after an initial programme created a community coalition or similar structure (a community level of analysis). This overview aims to examine sustainability in the context of individual level outcomes (i.e. health professional behaviour or change or proxy measures of change).

### Theories of sustainability

Healthcare professional behaviour change interventions have been developed to promote change in various professional groups, clinical practices and contexts. A variety of theoretical variables, based on psychological theories of behaviour change, have been identified as important in influencing healthcare professional behaviour [16]. These include aspects such as knowledge, skills, beliefs about capabilities, and motivation. Moreover, theories specific to healthcare professional behaviour have been developed and tested systematically, such as the Normalisation Process Theory [17], but this novel work has not yet led to the widespread development and testing of interventions. Any intervention might draw on a variety of routes to changing behaviour, but much of the healthcare professional intervention literature has lacked a clearly reported theoretical basis [18]. In the absence of explicit use of theory, a recent systematic review of healthcare professional interventions retrospectively examined the targets that interventions implicitly attempted to change [19]. The review found that several likely determinants of healthcare professional behaviour such as “social/professional role and identity”; “optimism”; “reinforcement”; “intentions” and “behavioural regulation” do not appear to have been considered in implementation interventions.

We will conduct an overview of Cochrane reviews of health professional behavioural change interventions. We will synthesise all high-quality review evidence relating to healthcare professional behaviour change and conduct systematic exploration of the evidence relating to long-term sustained outcomes. Overviews of reviews aim to provide a summary of evidence from more than one systematic review at a variety of different levels, including the combination of different outcomes [20], and can be particularly useful when several related intervention reviews have been completed. Overviews provide a succinct summary of the reviews relevant to a specific question and are particularly useful for decision makers such as clinicians and policy makers [20].

### Objectives

The objective of this study is to carry out an overview of reviews, based on the methods for Cochrane overviews [20], in order to report and synthesise the evidence of sustained change in healthcare professional behaviour resulting from interventions aimed at changing health professional behaviour. Where evidence exists, we will describe the extent of the evidence, the degree of sustained change (where “sustained change” is defined as measured change in professional behaviour more than 1 year after the start of the intervention) and the quality of the evidence.

## Methods

### Criteria for considering reviews for inclusion

Cochrane systematic reviews are generally of a better quality than non-Cochrane reviews [21–27]. The primary aim of a Cochrane overview should thus be to summarize multiple Cochrane intervention reviews [20].

We will therefore review all Cochrane reviews published in the Cochrane Database of Systematic Reviews (CDSR) with no date limit that meet the following inclusion criteria:

- Aim to synthesise data relating to change in group professional behaviour at more than 12 months follow-up
- Include randomised controlled trials (RCTs) or cluster randomised controlled trial (cRCTs). If a review includes quasi-randomised controlled trials (qRCTs) as well as RCTs, we will include data from the qRCTs if they have been pooled with data from the RCTs/cRCTs. However, if it is possible to extract data pertaining only to the RCTs/cRCTs, we will do this in preference to including data from qRCTs. In the event that we include evidence from qRCTs, we will highlight and discuss the implications of including this evidence
- Investigate intervention(s) for which a key aim is to change behaviour of any professionals in the delivery of a healthcare intervention, e.g. physicians, dentists, nurses, counsellors, clinical psychologists and allied healthcare professions involved in providing direct patient care
- Investigate the effect of behavioural change interventions on healthcare professional behaviour or patient outcomes which are proxy measures of healthcare professional behaviour change

We will exclude published reviews which:

- Only include studies aimed at changing the behaviour of patients, or a population of patients, and do not include an intervention targeting the behaviour of healthcare professionals. Where reviews include data on both healthcare professionals and patients, we will only include it if the authors have reported the data that specifically relates to healthcare professional outcomes.

The primary and secondary outcomes of interest to this overview are as follows:

### Primary outcome

- Sustained healthcare professional clinical behaviour (behaviour more than 1 year after the start of the intervention). This outcome can be measured by a range of measures, including (but not limited to):
- Rate/s of performing prevention, diagnosis and treatment behaviours (e.g. immunisation, blood pressure measurement, prescription, referral, hand washing)
- Assessment of adherence/fidelity to procedures or protocols

### Secondary outcomes

- Healthcare professional non-clinical behaviour, for example, attendance at education or training, professional meetings (sustained and less than 1 year after intervention).
- Patient level outcomes as proxy measures for healthcare professional behaviour, for example, number of infections as a measure of health professionals’ hand hygiene behaviour.

### Search methods for identification of reviews

Relevant reviews will be identified by hand searching the Cochrane Database of Systematic Reviews (CDSR) (*The Cochrane Library*, latest issue) by review group, for any published Cochrane reviews. We will only include published reviews. We will not include Cochrane overviews, as the objectives of this overview will differ from those of other overviews. However, we will handsearch reference lists of identified Cochrane overviews for reviews that might meet inclusion for the current overview. We will also contact authors of relevant protocols to ascertain intended completion dates.

### Data collection and analysis

#### Selection of reviews

One reviewer will download all of the reviews from the Cochrane (CDSR) library and will remove any obviously irrelevant titles. Two authors will independently review the abstracts of all remaining records, applying selection criteria to identify eligible reviews. Full papers will be obtained for all reviews considered potentially relevant by at least one reviewer and will be independently assessed for inclusion by two reviewers. Any disagreements between reviewers will be resolved through discussion, involving a third reviewer where necessary.

### Data extraction and management

Two reviewers will extract data independently. Any disagreements that arise will be resolved initially by discussion between the two reviewers, with assistance from a third reviewer, if necessary. We will use specifically designed data collection forms to extract and record the following:

- o *Overview of evidence*. We will synthesise key features of each review including details of the aims and rationale; date of last search; participants (healthcare professional population); interventions (brief description); comparisons; outcomes assessed >1 year from intervention; method of assessing quality of studies; and number of included studies and participants.
- o *Evidence of sustained change*. We will systematically identify any reviews which have measured our primary outcome at >1-year post intervention start.
- o *Intervention components*. For each of the reviews, we will describe the intervention components as coded by the review authors, including both intervention content and delivery components, and note any associations that authors report between intervention components and sustained effectiveness.

We will systematically synthesise the individual studies included within all identified reviews to explore whether any reviews covered the same studies. If overlap between reviews is identified, two overview authors will discuss the overlap with consideration of each review question, comparisons explored, date of the last search and key aspects of methodological quality (e.g. types of studies included, risk of bias assessment). We will use these details to reach agreement regarding which data from which review comparisons should be included within the overview. Any overlaps between included reviews or comparisons will be transparently reported.

For each relevant comparison reported in each included review, one overview author will systematically extract data on the risk of bias (as documented in the published review) ideally using the Cochrane risk of bias tool [28], of trials within the comparison and the results of any meta-analyses performed. These data will be checked by a second overview author with reference to the published review.

### Assessment of methodological quality of included reviews

#### Quality of included reviews

Two overview authors will independently assess the methodological quality of the included reviews, using the risk of bias in systematic reviews (ROBIS) tool [29] and present within tabular format. Any disagreements between overview authors will be resolved through discussion, involving a third author where necessary.

#### Quality of evidence in included reviews

We will report the quality of evidence of RCTs within included reviews, which have measures of sustained change. We will report quality of individual studies according to the review authors’ assessment using the Cochrane risk of bias tool [28].

For each included review which presents a statistical comparison (pooled analysis) for our primary outcome of sustained healthcare professional behaviour, we will assess and document the quality of the evidence synthesised within the reviews based on the criteria considered within the GRADE (Grading of Recommendations Assessment, Development and Evaluation) approach [30]. For each relevant comparison within an included review, we will classify our GRADE assessment, systematically assessing whether the quality of the evidence relating to each comparison is of:

- o high quality, when further research is very unlikely to change our confidence in the estimate of effect;
- o moderate quality, when further research is likely to have an important impact on our confidence in the estimate of effect and may change the estimate;
- o low quality, when further research is very likely to have an important impact on our confidence in the estimate of effect and is likely to change the estimate; or
- o very low quality, when we are very uncertain about the estimate.

Judgement of GRADE quality will be agreed through discussion involving at least three reviewers and involving additional reviewers where there is disagreement.

### Data synthesis

We will use a narrative synthesis of included reviews to summarise our findings. Where appropriate, included reviews will be categorised by type of intervention and/or outcome. The narrative synthesis will focus on the overall evidence for sustained effectiveness of interventions, and the association between study components and reported effectiveness, grouped according to the GRADE of the evidence.

### Registration

This manuscript is the public record of the current overview, and the protocol has therefore not been registered with PROSPERO.

## Discussion

The issue of sustained behaviour change is of paramount importance to decision makers, and currently, the long-term effects of interventions on health professional behaviour are disparate and difficult to access. This overview will therefore bring together into one accessible, comprehensive document, evidence from Cochrane reviews relating to the sustainability of health professional behaviour change, thus supporting policy makers with decision-making in this field.

## Abbreviations

BCT: Behaviour change techniques
cRCT: Cluster randomised controlled trial
GRADE: Grading of Recommendations, Assessment, Development and Evaluation
qRCT: Quasi-randomised controlled trial
RCT: Randomised controlled trials
ROBIS: Risk of bias in systematic reviews
